# Dual Targeting to Overcome Current Challenges in Multiple Myeloma CAR T-Cell Treatment

**DOI:** 10.3389/fonc.2020.01362

**Published:** 2020-08-05

**Authors:** Jort J. van der Schans, Niels W. C. J. van de Donk, Tuna Mutis

**Affiliations:** Department of Hematology, Cancer Center Amsterdam, Amsterdam UMC, Vrije Universiteit Amsterdam, Amsterdam, Netherlands

**Keywords:** chimeric antigen receptor T-cells, multiple myeloma, dual targeting, antigen escape, target choice, split signaling

## Abstract

In the era of highly promising novel targeted-immunotherapy strategies for multiple myeloma (MM), the first series of clinical trials with CAR T-cells targeting the plasma cell-specific B-cell maturation antigen (BCMA) have shown excellent response rates. In the long-term, however, MM appears to escape the therapy likely due to initial low and heterogeneous expression or downregulation of BCMA expression. Several other molecules targeted by CAR T-cells in MM are expressed at high levels on MM cells, but many of these attractive targets are also expressed on various, sometimes vital non-malignant cells, posing major risks for on-target, off-tumor side effects. CAR T-cell therapy for MM therefore faces two urgent challenges: (i) improving the efficacy of BCMA CAR T-cells and (ii) establishing a MM-selectivity even when CAR T-cells are directed against not entirely MM-specific target antigens. In this review, we will outline the current attempts to tackle these challenges, with a specific focus on how dual CAR targeting might be applied to tackle both issues.

## The Curative Potential of Immunotherapy in MM

Multiple myeloma (MM) is a progressive hematological malignancy resulting from the malignant outgrowth of monoclonal plasma cells in the bone marrow. As the second most common hematological malignancy, MM accounts for 2% of all cancer deaths in the U.S., as of 2019 (1). While the therapy of MM was for decades based on a number of alkylating agents, with melphalan being the prime choice, since the beginning of the century, treatment options for MM patients have been significantly improved by successful applications of immunomodulatory drugs, proteasome inhibitors, histone deacetylases, and by monoclonal antibodies (2). Although each of these novel therapies significantly improved the outcome of patients, MM still remains an incurable disease for the majority of patients. Most, if not all patients develop resistance, even to triple combination therapies. The life expectancy of multi-drug resistant patients is very short, urging for more powerful, potentially curative approaches.

For many decades, it has consistently been observed that anti-MM activity can be achieved due to the donor T-cell mediated graft-versus-myeloma effect after allogeneic stem cell transplantation. Long-term survival in a proportion of patients, thanks to allogeneic stem cell transplantation, clearly illustrates that the powerful cytotoxic machinery of T-cells is capable of eradicating multi-drug resistant MM cells. Therefore, T-cell based therapies, provided that they are broadly applicable, affordable, and can be made selective for targeting MM cells, could be highly beneficial to achieve the ultimate goal of cure in MM. Over the past decade, autologous chimeric antigen receptor engineered T-cells (CAR T-cells) targeting tumor-associated lineage antigens, such as the B-cell specific CD19 antigen, emerged as a successful therapeutic modality, with very high success rates in B-cell malignancies (3). Clinical trials executed with CD19 CAR T-cells resulted in the effective elimination of malignant as well as healthy B-cells (4–6). The striking remissions were not compromised with extreme unmanageable side effects due to elimination of healthy B-cells. Consequently, today two CD19-based CAR T-cell therapies have been approved by FDA as a therapeutic modality for B-cell acute lymphoblastic leukemia and diffuse large B-cell lymphoma patients.

## BCMA: The Ideal MM Car Target?

Prompted by the success of CD19 CAR T-cells, several CAR T-cell therapies are also being developed for MM, among which, major efforts have been devoted to CAR T-cells targeting the B-cell maturation antigen, BCMA (7–11). The choice for BCMA, already discovered in 1992 as a translocation counterpart with the IL2 gene in a patient with T-cell lymphoma, as a prime target for CAR T-cells was obvious, because BCMA is normally expressed solely on a subset of mature B-cells and on antibody-producing plasma cells, including their malignant counterparts, the MM cells. BCMA is therefore a safe target, because other than plasma cell aplasia, no risk of adverse events resulting from on target, off tumor effects are expected when BCMA is targeted by CAR T-cells. Furthermore, BCMA was shown to be important in proliferation and survival of MM cells, another important feature, substantiating the value of BCMA as a suitable target for immunotherapy (12).

On the other hand, several studies found the expression of BCMA in MM cells to be heterogeneous, with extremely low expression levels in some patients (13). Furthermore, in a recent genome-wide CRISPR gene-editing study, BCMA was not found among more than 90 genes essential for MM (14). These are properties which do not fit with the description of the “ideal” target for immunotherapy (15, 16).

The first in man study with BCMA-targeted CAR T-cells was performed by the National Cancer Institute (NCI) in heavily pretreated patients. At the highest dose-level tested, the overall response rate [≥partial response (PR)] was 81% in patients with a median of 10 prior lines of therapy (17). At this moment there are two CAR T-cell products that are very advanced in terms of clinical testing and both are currently being reviewed by regulatory authorities for their application in patients with advanced MM. This includes idecabtagene vicleucel (ide-cel; bb2121), which expresses a murine BCMA-targeting single-chain variable fragment with 4-1BB costimulatory motif. In the phase 1 study with ide-cel, at least PR was achieved in 85% [including complete response (CR) in 45%] of heavily pretreated patients with a median 7 prior lines of therapy. The median PFS in patients who received ≥150 × 10^6^ CAR T-cells was 11.8 months (8). The second CAR T-cell product, that is submitted to regulatory authorities, is JNJ-4528 (LCAR-B38M), which is a 4-1BB-based CAR T-cell therapy with 2 BCMA-targeting domains, which confers high avidity binding. JNJ-4528 was evaluated in the CARTITUDE-1 study in patients exposed to immunomodulatory drugs, proteasome inhibitors and CD38 antibodies (18). Preliminary results of the first 29 patients treated with JNJ-4528 (target dose: 0.75 × 10^6^ CAR T-cells/kg) demonstrated a 100% response rate with CR in 69%. With a short median follow-up of 6 months, 27 of 29 patients remained progression-free. The same product was also evaluated in the LEGEND-2 study in a less heavily pretreated patient population in China. Results from one site showed that at least PR was obtained in 88% of patients (CR in 74%) with a median of 3 prior lines of therapy (10, 19). The median PFS was 19.9 months (28.2 months for patients with CR) (10). In these studies toxicity consisted of cytokine-release syndrome, cytopenias, and infections, while neurological toxicity was less frequent than observed in studies with CD19 CAR T-cells.

Altogether, these studies demonstrate that independent of which CAR T-cell construct has been used, the early clinical responses to BCMA CAR T-cells are extremely well, but, in studies with sufficient follow-up, many patients have short remission duration, and show relapse after BCMA-targeted CAR T therapy (8, 10, 20). Relapsed patients show a lack of CAR T-cell *in vivo* persistence and BCMA-low, or in some cases BCMA-negative, disease which may be due to the low, and heterogeneous expression or downregulation of BCMA from the cell surface (17, 21–24). Therefore, preventing the escape of MM cells from BCMA CAR T-cell therapy is currently an important challenge. For instance, improving CAR design to prevent T-cell exhaustion, preventing rapid development of effector memory T-cell phenotype by introducing CARs into naïve or central memory T-cells or avoiding tonic CAR signaling could further improve patient outcome (25, 26). As an alternative, there are also several other potential target molecules expressed at high and homogenous levels on the surface of MM cells. As outlined in the next section, CAR T-cells against these molecules are also being developed and tested in preclinical settings or even in clinical trials. Nonetheless, all of these “alternative” targets are also expressed on some various non-malignant cells, posing potential risks for on-target, off-tumor side effects. Hence, establishing a MM-specific effect by targeting MM-associated but not entirely MM-specific target antigens is another potentially important challenge of CAR T-cell therapy in MM. Below we will first outline the advantages and possible disadvantages of several alternative target antigens for CAR T-cells in MM and will focus on how except GPR5Cection g also developed and testedapym for CAR-T cells and specific modalities of dual-CAR targeting can exploit these alternative targets to offer solutions for the current challenges of CAR T-cell therapy in MM.

## MM Targets Other Than BCMA

G-Protein Coupled Receptor 5D (GPRC5D) has recently been identified as another potential MM target, because this antigen is expressed on malignant MM cells at high levels, independent of BCMA distribution, and only in low levels on B cells, healthy plasma cells and hair follicles (27, 28). Its function, ligand and its role in MM development is not yet known, but the enhanced expression on MM cells compared to healthy plasma cells indicates a role in malignancy. Targeting this largely MM-specific molecule with CD3/GPRC5D bispecific antibodies and CAR T-cells in preclinical settings has already shown promising results and ongoing clinical studies will expose its suitability as a MM-target (27, 29). Except BCMA, and perhaps GPRC5D, no other plasma cell or MM cell-specific surface antigens have been discovered so far. Though, MM cells express many other attractive target antigens, of which CD138, CD38, and SLAMF7/CS1 are the most prominent ones. All these antigens are highly expressed on MM cells. While SLAMF7 expression might be slightly reduced upon disease progression, CD38 expression is generally unaffected at different disease stages and CD138 displays an even higher expression on MM cells from patients with refractory and progressive disease (30–33). All these antigens are however also expressed on other tissues. The high expression of CD138 on normal tissues (i.e., squamous epithelium, hepatocytes, goblet, and columnar cells of gastrointestinal tract) suggests that its sole targeting can be associated with on target, off tumor side effects. Indeed, targeting CD138 with an antibody drug conjugate (BT062) induced skin and mucosal toxicity, although such side effects have not been seen in a small pilot trial with CD138 CAR T-cells (34, 35). The expression of CD38 and SLAMF7 on non-malignant hematopoietic cells, such as T-cells, B-cells, NK-cells, macrophages, dendritic cells is lower as compared to MM cells (30, 36). CD38 is also expressed in other tissues, such as in lung smooth muscle cells and in Purkinje cells but at most at intermediate levels, thus generating a clear differential expression window that can be exploited by carefully designed targeted therapies. This has been shown with antibody targeting of CD38 with daratumumab and isatixumab, and of SLAMF7 with elotuzumab, which were well-tolerated in patients with newly diagnosed and relapsed/refractory MM (37–39). Encouraged by these results, these molecules were also targeted with CAR T-cells. In preclinical studies, CAR T-cells generated against SLAMF7 using the antibody elotuzumab as a binding domain are highly effective but they cause lysis of SLAMF7^+^ fractions of T-, B-, and NK-cells, requiring precautions such as the inclusion of suicide genes, in the design of ongoing clinical studies (40) (NCT03958656, NCT03710421). Similarly, CD38 CAR T-cells generated from high affinity antibodies effectively eliminate MM cells but also kill CD38^+^ non-malignant cells. Fortunately, it is possible to generate CD38 CAR T-cells with optimized lower-affinities to efficiently eliminate MM cells without any undesired cytotoxic activity against normal hematopoietic cells (41). The safety profile and efficacy of CD38 CAR T-cells (CAR2 Anti-CD38 A2 CAR T-Cells) generated from an apparently similar low-affinity antibody is currently being tested in a clinical trial (NCT03464916), with no reported interim results yet. Besides the above mentioned targets, many other MM-associated, but not entirely plasma cell, or MM-specific targets, such as NKG2D, CD56, Lewis-x, CD44v6 are evaluated for CAR T-cell therapy (42–44), with currently no reported outcomes of the latter three (NCT04097301, NCT03473496, NCT01716364). NKG2D CAR T-cells obtained no clinical response or side effects, which may be because CAR T-cells were infused without prior lympho-depletion, or, as found in preclinical work, NKG2D CAR NK-cells rather than T-cells were effective in eliminating MM cells (NCT02203825) (42, 45).

## Dual Car T-Cell Targeting to Improve Efficacy

**Figure 1:**
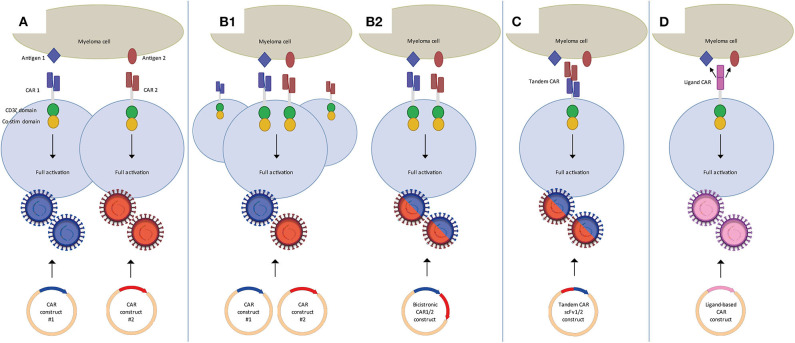
Dual CAR strategies to improve efficacy of CAR T-cell MM therapy. **(A)** Mixture of two pools of single CAR T-cells in which each pool targets a different antigen. These can be co-infused or sequentially infused into a patient. (B) Two CARs targeting distinct antigens expressed on one T-cell through the use of co-transduction **(B1)** or a bicistronic vector **(B2)**. Binding of either one of the CARs is sufficient to activate the T-cell. **(C)** Tandem CAR T-cells, which contain two distinct binding domains linked to one receptor. Binding of either one of the domains is sufficient to activate the T-cell. **(D)** Ligand-based CAR T-cells, containing a ligand as binding domain that can recognize more than one antigen. Binding of either one of the antigens is sufficient to activate the T-cell.

**Figure 2:**
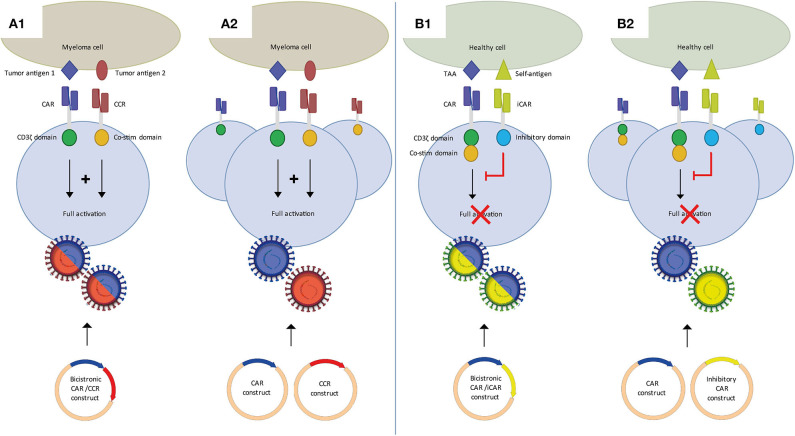
Dual CAR strategies to improve specificity of CAR T-cell MM therapy. (A) Split CAR T-cells, in which activation through CD3ζ and co-stimulation are split over a first generation CAR and a chimeric co-stimulatory CAR (CCR), each targeting distinct tumor antigens. Binding of both CARs is required for full T-cell activation. Split CAR T-cells can be generated using a bicistronic vector **(A1)** or two separate CAR and CCR vectors **(A2)**. (B) An inhibitory CAR (iCAR) binding a self-antigen inhibits T-cell activation of a second CAR targeting a tumor-associated antigen (TAA). In the absence of a self-antigen it functions as a conventional single CAR T-cell. CAR/iCAR combinations can also be made using a bicistronic vector **(B1)** or two separate CAR and iCAR vectors **(B2)**.

As illustrated above for CD38 CAR T-cells, clinical translation of strategies targeting MM-associated, but not entirely MM-specific antigens, may require various adjustments to increase their MM cell selectivity. These alternative target antigens may also be carefully combined with BCMA CAR T-cells in dual CAR T-cell targeting strategies to prevent MM escape and improve the overall efficacy of CAR T-cells (Figure 1) or even to establish MM-specificity by applying split signaling approaches (Figure 2). The most interesting approaches that are aiming at improving the efficacy of CAR T-cells are combination of BCMA CARs with CD19, CD38, SLAMF7, and GPRC5D CARs either by co-infusion of two pools of T-cells that express distinct CARs (Figure 1A) or by infusion of a single T-cell pool in which each T-cell expresses two distinct CARs (Figure 1B). All these strategies have specific advantages and disadvantages. For instance, dual-targeting by distinct CAR T-cells has the advantage that CAR expression in both CAR T-cell products can be separately controlled. Moreover, this strategy allows the sequential administration of CAR T-cells, which may decrease the risk of severe cytokine release syndrome (CRS). With this idea, in two recently published clinical trials BCMA/CD19 dual CAR T-cell targeting was achieved using two pools of single CAR transduced T-cells in newly diagnosed as well as in relapsed refractory patients (NCT03706547, NCT03767725). Patients received sequential administration of CAR T-cells in order to decrease the risk of aggravated cytokine release syndrome (CRS). In both studies the patients developed manageable CRS but no neurological toxicity. The clinical responses of evaluable patients suggested that BCMA-CD19 CAR T-cell sequential combination could improve the clinical response. Nonetheless, since these studies lacked a control group (i.e., single arm) it will be highly important to evaluate the results of a recently initiated study (NCT03549442) which addresses whether dual targeting of BCMA/CD19 is more effective and equally safe as compared to BCMA targeting only, besides comparing co-infusion and sequential infusion of BCMA and CD19 CAR T-cells.

Since generation of two different batches of CAR T-cells may have important negative impacts on the feasibility and the costs of CAR T-cell production several other attempts are done with T-cells that are simultaneously transduced with two different, fully functional second-generation CARs (Figure 1B). Technically, the dual CAR specificity of a single T-cell can be obtained either by simultaneous transduction of two separate CAR constructs into T-cells or using a single bicistronic vector containing two separate CAR constructs. Achieving high transduction efficiencies for dual CARs through a bicistronic vector might be more challenging because of the size of the construct (46, 47), but this assures that both CARs are expressed in all transduced cells, and at the same density level. This is challenging in a dual transduction strategy using two separate CAR constructs. Dual transduction usually results in a mixture of cells expressing single as well as dual transduced CAR T cells, requiring further sorting to obtain a pure product. Furthermore it is difficult to express both CARs expressed at similar density levels with the dual transduction strategy.

Dual CAR T-cells targeting the combinations of BCMA/CD19, BCMA/SLAMF7 constructed with bicistronic vectors are now being evaluated in clinical trials (48) (NCT04156269, NCT04162353). Several other clinical trials with unspecified dual targeting approaches test combinations that include BCMA/CD38, BCMA/NY-ESO1, and CD38/CD19 (NCT03125577, NCT03767751, NCT03473496, NCT03271632, NCT03638206). As presented at ASH2019, BCMA/GPRC5D dual CAR T-cells are being evaluated pre-clinically, either co-infused, co-transduced or made with a bicistronic vector (49).

Alternatively, dual target specificity can be achieved by insertion of bispecific tandem CAR constructs (Figure 1C). Tandem CAR T-cells comprise a single second, or higher, generation CAR with two distinct scFv's. Several tandem CARs have been studied for the treatment of B-cell malignancies, targeting the combinations of CD19/CD20 and CD19/CD22 (50, 51). So far, for MM no results on tandem CARs targeting two distinct antigens have been published. However, the LCAR B38M trial uses a CAR that targets two distinct epitopes of BCMA, claiming increased recognition and efficacy (10, 20).

The generation of ligand-based CARs that can target two separate antigens is yet another way to achieve dual target specificity (Figure 1D). The APRIL-CAR is a typical example of a ligand-based CAR. In this strategy, the extracellular domain of APRIL, the natural ligand of BCMA, is constructed in a CAR, instead of using an antibody derived scFv. The major advantage of using APRIL as binding moiety is that, besides BCMA, it also recognizes transmembrane activator, and calcium-modulator and cyclophilin ligand (TACI). Therefore, this CAR can target two MM-associated antigens simultaneously. After successful preclinical evaluation, APRIL CAR T-cells were being tested in a clinical trial, but the trial was recently terminated because of disappointing results (52) (NCT03287804). Therefore, although the idea is attractive, APRIL-based CAR T-cells will need further optimization. Schmidts et al. (53) showed such an optimization, by changing the monomeric structure of APRIL to a trimeric structure, which increased efficacy of APRIL-based CAR T-cells in a pre-clinical study.

## Dual Car T-Cells to Improve MM-Specificity

Alternatively, and perhaps more appealing, dual antigen targeting by CAR T-cells can be exploited to establish MM-specificity of CAR T-cell therapy by the application of split dual CAR T-cell strategy. This concept, for the first time shown by Kloss et al. (54), is based on the fundamental concept that T-cells require two distinct signals to become fully activated. In the split-dual CAR technology, the primary activation, and the co-stimulation signals for T-cells are split into two separately expressed CARs that are directed against two carefully selected antigens, which are individually not tumor-specific, but in combination display tumor-specific expression. Splitting the first and co-stimulatory T-cell activation signals will thus enable tumor specificity, because dual CAR-transduced T-cells can only be fully activated if both CARs simultaneously engage their targets on the tumor cells but not if they recognize only one of the antigens on the normal tissues (Figure 2A). Important was to lower the affinity of the CAR linked to CD3ζ to diminish the possibility of activation through this CAR alone. Until now, four groups have shown the feasibility of using split signaling CAR T-cells in different types of solid tumors and AML (55–58). While the main purpose of split-dual CAR T-cells is to increase specificity, split-dual CARs can also improve the avidity of CAR T-cell to the tumor target. Since BCMA expression is not expressed on healthy cells other than plasma cells, it may not be necessary to use split signaling to increase specificity of BCMA CAR T-cells. However, it may be beneficial and open new possibilities of using MM antigens that are not restricted to plasma cells and myeloma cells, especially antigens that are highly expressed on MM cells, but lower on healthy cells, such as CD138, CD38, and SLAMF7.

Finally, another form of a dual CAR design to improve specificity uses inhibitory CARs (Figure 2B). This approach was first described by Fedorov et al. (59). A conventional second generation CAR is combined with an inhibitory CAR (iCAR), containing an scFv binding domain linked to an inhibitory cytoplasmic domain, such as PD-1. Combining a non-specific MM target with an iCAR targeting a self-antigen may improve specificity and therefore safety of MM CAR therapy. Both split-signaling CAR T-cells and dual CAR/iCAR T-cells can be generated through co-transduction of two separate vectors or the use of a bicistronic vector, with accompanying advantages and disadvantages as discussed previously.

## Conclusion

Dual or multi-targeting is a promising tool both to tackle target antigen loss or downregulation and to allow the use of MM-associated, but not specific, target antigens. There are several options in producing dual CAR T-cells and obtaining dual CAR targeting, of which most are being explored in the MM setting. Importantly, at present, there is only little research done on comparing different dual CAR targeting approaches in efficacy and persistence. In pre-clinical work, Hamieh et al. (22) found that co-infused CD19 and CD22 CAR T-cells were superior in preventing relapse due to low target antigen presence, to the same CAR T-cells sequentially infused. Fernandez De Larrea et al. (49) compared a 1:1 mix of BCMA and GPCR5D CAR T-cells, to a single bicistronic BCMA/GPCR5D construct and a single CAR with BCMA and GPCR5D scFv's in tandem and found the bicistronic construct to be superior when tested in a sub-therapeutic dose. Similarly, Ruella et al. (60) found that a dual CD19/CD123 CAR T-cells created with a bicistronic vector outperformed pooled CD19 and CD123 CAR T-cells. However, using different target antigens and CARs may very well change this outcome and more research is needed to determine which approach is best suited in a specific situation.

As briefly discussed earlier, besides relapse related to down-regulation, there are likely other mechanism of resistance in MM CAR T-cell treatment. Lack of CAR T-cell persistence despite target antigen presence and suppression of CAR T-cells by inhibitory molecules, such as PD-L1, or immunosuppressive cells, such as myeloid-derived suppressor cells (MDSCs), may also play a role. Persistency may be augmented by using optimal co-stimulatory domains and preventing tonic signaling (25, 61). Moreover, the phenotype of the T-cells appears to be important for persistence and several studies are exploring how to optimize this (26, 62). Split signaling strategies may open possibilities of using less specific MM target antigens, but by requiring two target antigens, antigen escape may increase since loss of one of the antigens is already sufficient to abrogate CAR T-cell activation. It is therefore important to target antigens that are implicated in disease progression and are expressed at high levels, as this may reduce the likelihood that MM cells decrease or lose antigen expression and to exploit the differential expression of target antigens on healthy and malignant tissues optimizing CAR affinity.

## Author Contributions

JS and TM reviewed the literature, participated in the design of the manuscript, and wrote the paper. ND revised the manuscript substantially for important intellectual content. All authors contributed to the article and approved the submitted version.

## Conflict of Interest

TM has received research support from Janssen Pharmaceuticals, Genmab, Takeda, Onkimmune, and Gadeta. ND has received research support from Janssen Pharmaceuticals, AMGEN, Celgene, Novartis, and BMS and serves in advisory boards for Janssen Pharmaceuticals, AMGEN, Celgene, BMS, Takeda, Roche, Novartis, Bayer, and Servier. The remaining author declares that the research was conducted in the absence of any commercial or financial relationships that could be construed as a potential conflict of interest.
